# Mathematical Modeling of Breast Tumor Destruction Using Fast Heating during Radiofrequency Ablation

**DOI:** 10.3390/ma13010136

**Published:** 2019-12-28

**Authors:** Marek Paruch

**Affiliations:** Department of Computational Mechanics and Engineering, Silesian University of Technology, 44-100 Gliwice, Poland; marek.paruch@polsl.pl

**Keywords:** Arrhenius scheme, bioheat transfer, breast cancer, electric field, finite element method, hyperthermia, mathematical modeling, numerical methods, radiofrequency ablation, thermal ablation

## Abstract

In oncology, hyperthermia is understood as a planned, controlled technique of heating cancerous changes in order to destroy their cells or stop their growth. In clinical practice, hyperthermia is used in combination with radiotherapy, chemotherapy, or immunological therapy. During the hyperthermia, the tissue is typically exposed to a temperature in the range of 40–45 °C, the exception is thermoablation, during which the temperatures reach much higher values. Thermoablation is characterized by the use of high temperatures up to 90 °C. The electrode using the radiofrequency is inserted into the central area of the tumor. Interstitial thermoablation is used to treat, among others, breast and brain cancer. The therapy consists of inducing coagulation necrosis in an area that is heated to very high temperatures. Mathematical modeling is based on the use of a coupled thermo-electric model, in which the electric field is described by means of the Laplace equation, while the temperature field is based on the Pennes equation. Coupling occurs at the level of the additional source function in the Pennes equation. The temperature field obtained in this way makes it possible to calculate the Arrhenius integral as a determinant of the destruction of biological tissue. As a result of numerical calculations regarding the temperature field and the Arrhenius integral, it can be concluded that, with the help of numerical tools and mathematical modeling, one can simulate the process of destroying cancerous tissue.

## 1. Introduction

Cancer diseases are one of the main causes of death in both the societies of highly developed and low developed countries. In recent years, their intensity has increased particularly, which caused cancer to become not only a serious health problem, but also a social and economic problem [1].

Breast cancer is the second most common malignancy in the world, right after lung cancer. In the female population, it is the most frequently occurring malignant tumor. According to data from the World Health Organization, in 2018, 2.1 million new cases of this disease were diagnosed, which is 11.6% of all cancers in women [2]. 

Breast cancer is a malignant tumor arising from glandular tissue cells that develops locally in the breast and provides metastases to the lymph nodes and internal organs (e.g., lungs, liver, bones, and the brain). This cancer is the most common malignancy in women. It accounts for about 23% of all cases of malignant tumors in women and about 15% of deaths for this reason. It is estimated that 2.1 million women are diagnosed with breast cancer every year around the world, and about 620,000 die of it [2,3].

Breast cancer is the biggest oncology problem in developed countries; it is also a growing problem in developing countries. An example is the comparison of the number of patients and deaths: in developed countries, there are slightly more than half of the cases (50.1%), while the majority of deaths are recorded in less developed countries (58.7%) [4,5].

Medicine increasingly uses the possibility of performing treatments with methods of limited invasiveness (so-called minimally invasive procedures). One of such techniques is thermoablation, i.e., destruction of pathological tissues by means of high temperature. It has a special application in oncology. Based on the energy sources and their delivery, the thermal ablative techniques can be classified into five major categories, namely, radiofrequency ablation (RFA), laser ablation (LA), microwave ablation (MWA), cryoablation, and high-intensity focused ultrasound (HIFU) ablation [6]. An interesting review of the literature on thermoablation of a breast tumor using the above-mentioned methods can be found, among others, in [7].

The most common thermoablation, using electromagnetic waves, consists of inserting the electrode in the form of a needle into the central site of the tumor. This can be done traditionally by opening the abdominal cavity (e.g., in the case of a liver tumor) or through the skin during laparotomy or laparoscopy. The process is carried out under the control of ultrasonography, which is widely recognized as the best examination in the imaging of the surgery during its performance, or under the control of a computed tomography.

The needle-electrode used in this technique transmits high frequency current (300–500 kHz, which corresponds to the range of long and medium radio waves, usually 460 kHz). In this way, thermal energy is generated. The high electric intensity, resulting in the highest temperature, is at the tip of the electrode. The high temperature obtained in this way allows carbonization of carbohydrates contained in the tissues. The degree of destruction depends on the temperature obtained and the time of current flow. Temperature between 50 and 100 °C causes coagulation necrosis. Temperatures up to 45–50 °C induce necrosis and contribute to the destruction of cancer tissue due to dehydration, thickening, and denaturation of intracellular proteins, as well as destroying cell membranes. Destruction of the lesion with a diameter of approx. 3 cm, and therefore optimal in terms of the size of the area undergoing treatment, requires the influence of current in 10 to 15 min [8].

The beginning of treatment with a high temperature is at the end of the 19th century. The breakthrough came in 1891, when Jacques-Arsène d’Arsonval discovered that the flow of high-frequency current through the tissues leads to heat in them, without causing muscle contraction. In 1990, the destruction of liver tumors in animals was initiated by placing inside the electrode connected to the power generator. In the case of human liver tumors, thermoablation entered clinical practice in 1995, and, five years later, it was officially approved in the United States by the Federal Drug Administration (FDA) for use in cases of unresectable liver tumors [9].

## 2. Materials and Methods 

### 2.1. Introduction

In the case of electric field impact, numerical modeling consists of solving a coupled problem, and, more specifically, analysis of electro-thermal coupling. A tissue model is selected for the mathematical description of heat transfer, namely the classic Pennes model [10] (the most commonly used mathematical model describing the process of heat transfer in living organisms, on a macro scale, which is commonly used in particular to predict temperature changes during hyperthermia, or hypothermia treatments, i.e., broadly understood thermotherapy, e.g., [11,12,13,14,15,16]). The Pennes equation is actually a Fourier heat conduction equation with an additional source component that takes into account the global heat flow effect between blood and the surrounding tissue. In 1948, Pennes [10] proposed a model of the heat transfer in which the presence of a system of small blood vessels and metabolic processes occurring in tissues was taken into account by adding appropriate source components to the Fourier equation. The source function proposed by Pennes is the sum of two components called the perfusion source and the metabolic source. The idea of this equation is that blood at a temperature equal to blood temperature in the aorta flows through the tissue at a certain speed, achieving thermal balance in it, and then accumulates in the veins. The rate of blood perfusion is therefore a key parameter in the calculation of the bioheat transfer. Pennes differential equation is presented in Section 2.3. In turn, the mathematical description of the electric field is based on the Laplace equation. The Laplace differential equation is a second order linear partial differential equation. The name of the equation comes from the name of Pierre Simon de Laplace, who formulated it in the 18th century. The Laplace equation describes many physical processes. In a typical situation, the function contained in it is interpreted as a density of a certain magnitude (e.g., chemical concentration, temperature, potential). This equation expresses the following property of the potential field: divergence of the vector potential field (i.e., potential gradient), in the absence of the source is equal to zero. The Laplace equation is a special case of the Poisson equation expressing an analogous relationship in the case of the field sources. Therefore, it describes many processes occurring in nature, e.g., in electrostatics, electrostatic potential in the absence of electric charge, in thermodynamics, it describes the steady-state heat transfer process, in fluid mechanics, it describes the steady flow of liquids, in electrodynamics, it describes current flow in extended media, and, in mechanics, it describes deformations of elastic material. Simplification involving the use of the above equation to the mathematical description of the electric field is related to the following assumption. During medical procedures based on radiofrequency ablation caused by the electric field, a current with frequencies in the range of tens to several hundred kHz is induced. In such a frequency range, the electromagnetic wave length is much larger than the depth of the human body; therefore, the current flow is via electrical conductivity and can be analyzed as a so-called quasi-static formulation, which allows coupling the electrical and thermal conductivity problem [8,17,18,19,20,21,22]. To determine the internal heat source resulting from the influence of the electric field, it is necessary to know the intensity of the electric field, which is dependent on the electric conductivity coefficient.

The coupling of these two problems takes place by means of the weak formulation, which consists of taking into account the source function resulting from the interaction of the electric field in the Pennes equation, describing the heat transfer in the biological tissue.

Besides determining the temperature field in the biological tissue affected by the tumor and subjected to external thermal action, it is also necessary to estimate the degree of tissue destruction under the influence of high temperature. In the literature, e.g., [15,20,23,24], we can find information that the temperature above 45 degrees of Celsius is the so-called ablation temperature, i.e., causing irreversible destruction of biological tissue (the duration of the heating process is also important). It is obvious that tissue destruction is reflected in the values of its parameters, which may assume nonlinear values depending on the processes involved. Very often, the perfusion coefficient is treated as an indicator of the main tissue damage. A simple solution is the dependence of the perfusion coefficient on the temperature, assuming that exceeding the temperature at the point under consideration above 45 degrees of Celsius causes the disappearance of blood perfusion. In the literature, we can find relations describing the relationship between temperature and perfusion, as well as between perfusion and tissue destruction. One such method is to determine the value of the Arrhenius integral, which will be discussed in more detail in the next section. At this point, however, several facts should be presented. The "Arrhenius Integral" or "Arrhenius Equation" was physical justification and interpretation in 1889 by Svante Arrhenius, a Swedish chemist. Arrhenius performed experiments that correlated chemical reaction rate constants with temperature. After observing that many chemical reaction rates depended on the temperature, Arrhenius developed this equation to characterize the temperature-dependent reactions. The Arrhenius equation is successfully used not only in estimating the degree of destruction of biological tissue, as is the case in this article, but also in issues of physical chemistry, e.g., [25], or issues related to the analysis of the behavior of metals or alloys during high-temperature deformation, e.g., [26], and I suppose in many other problems.

### 2.2. Breast Modeling

**Figure 1:**
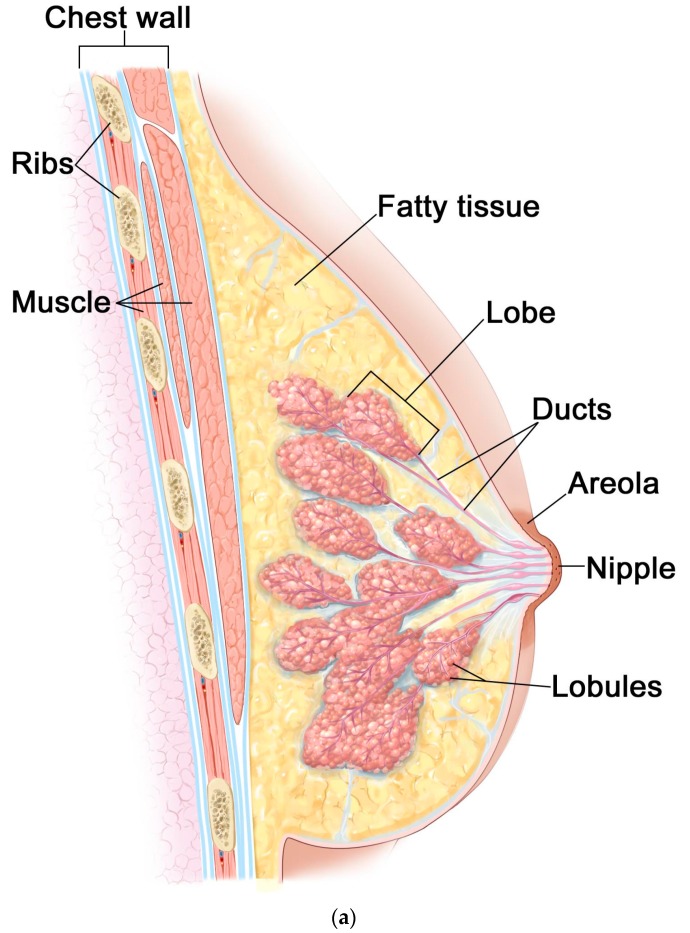

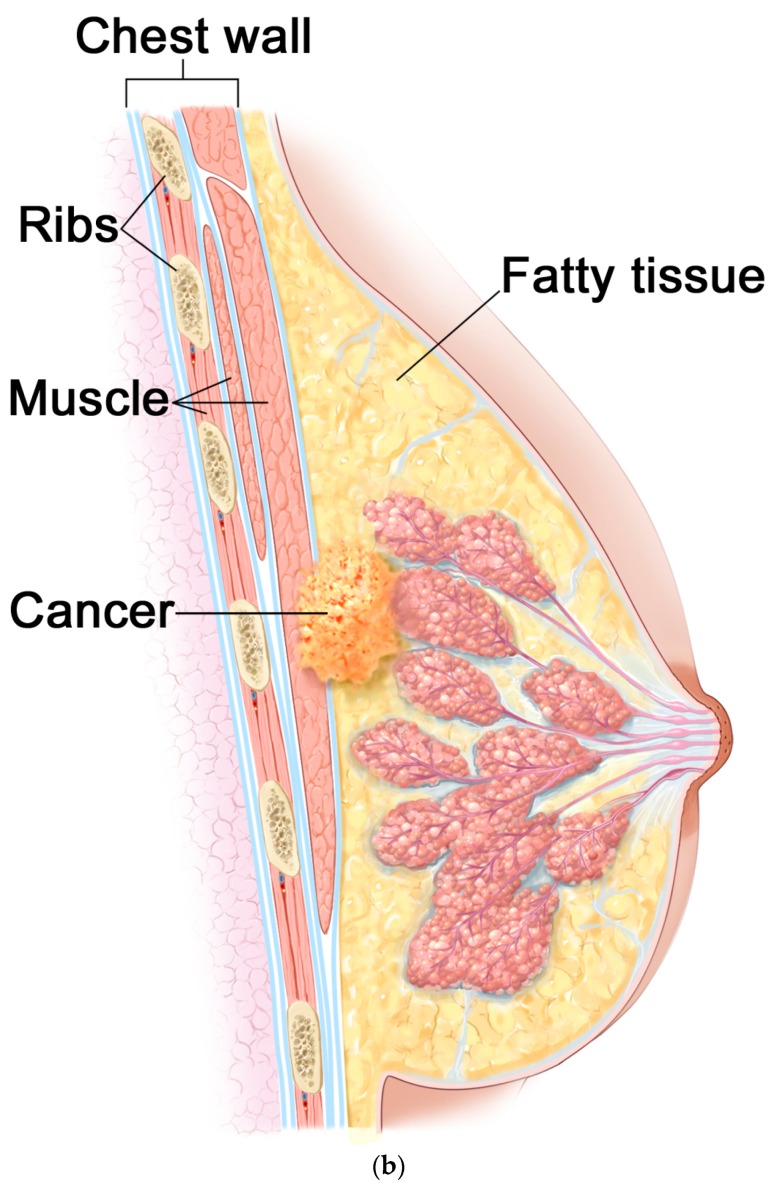
Anatomy of the female breast: (**a**) healthy breast; (**b**) breast with cancer. Reproduced by permission from Terese Winslow; Adapted from [27,28].

Both men and women have breast glands, but there are many more tissues in the women breast. Each breast is located on the chest muscle, called the pectoral muscle. The feminine breast covers a relatively large area. It stretches from the place just below the collarbone, to the armpit, and across the bridge. They are made up of lobules (milk-producing glands) and ducts (tubes that carry milk to the nipple). These are surrounded by glandular, muscular, and fatty tissue (cf. Figure 1a [27]).

The breast is a very complex and largest human gland. During human life, it undergoes more changes than any other part of the human body—from birth through puberty, pregnancy and breastfeeding, until menopause. It contains from 15 to 20 glands called lobes. The lobes are connected to the nipple with several tubes called ducts that carry milk to the nipple. The breast consists of lymph nodes and vessels that allow the transmission of lymph fluid and white blood cells. The rest is mainly fatty tissue. The breast, like any other part of the body, is made up of billions of microscopic cells. These cells usually reproduce in an orderly way—new healthy cells divide and then replace those that have died. However, sometimes, it happens that cells develop abnormally (mutations appear). This happens when the genes responsible for checking that cells are reproducing correctly do not detect changes or mutations. In this situation, pathological cells divide and multiply, and their growth is sometimes quite fast. At this stage, a growth may not be cancerous. At this point, we can deal with the so-called "non-invasive tumor", which is located in the lobe or the duct. When the tumor can infect the surrounding tissue, it is treated as cancerous. These types of cancers require immediate treatment because, if they continue to grow and propagate, they will be life-threatening. Breast cancer most often develops in glandular tissue (cf. Figure 1b [28]).

**Figure 2:**
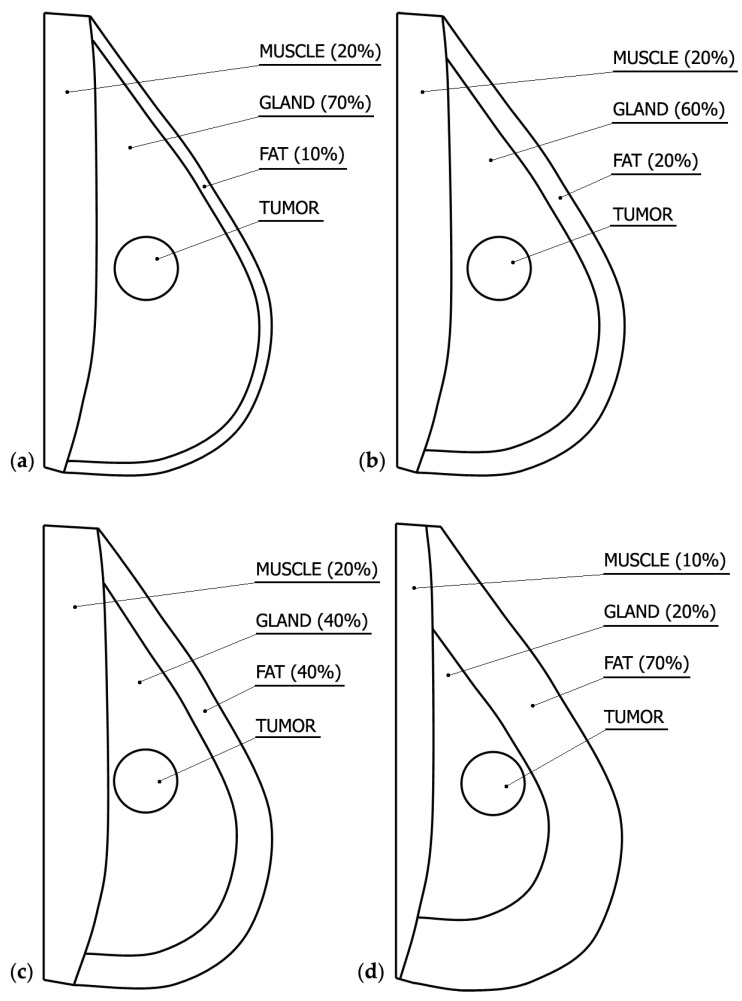
Cross-sectional view of the developed breast models (**a**) extremely dense (ED); (**b**) heterogeneously dense (HD); (**c**) scattered fibro-glandular (SFG); (**d**) predominantly fatty (PF).

For the purpose of numerical calculations, a multi-layered breast geometric model is prepared. Among the various configurations, the following types of models can be distinguished: extremely dense (ED), heterogeneously dense (HD), scattered fibro-glandular (SFG), and predominantly fatty (PF) [29,30]. Figure 2 presents the cross-sectional view of the developed breast models with the percentage of tissue compositions for each layer.

Because the number of breast cancer cases among women increases after the age of 35, the HD model was selected for analysis (see: Figure 2b), whose volume share of individual layers is appropriate for this age. Following [30], the percentage of layers of HD model can be determined at the level: muscle layer—20%, glandular layer—60%, and fat layer—20%.

### 2.3. Mathematical Modeling of Electric and Temperature Fields

Because the wavelength of the RF current in tissues is much greater that depth of a human body, the quasi-static electric field approximation is applied. Using the quasi-static formulation, the electric field intensity **E** (V/m) inside the biological tissue for 2D problem can be calculated as follows:

(1) $$\begin{array}{l} E_{e}\left(x_{1},x_{2}\right)=-\nabla \varphi_{e}\left(x_{1},x_{2}\right)= \end{array}-{\left[\begin{array}{cc} \frac{\partial \varphi_{e}\left(x_{1},x_{2}\right)}{\partial x_{1}} & \frac{\partial \varphi_{e}\left(x_{1},x_{2}\right)}{\partial x_{2}} \end{array}\right]}^{T}$$

where subscript *e* denotes the sub-regions of breast, electrode and trocar (i.e., 1—muscle, 2—gland, 3—fat, 4—tumor, 5—electrode, 6—trocar), {*x*_1_, *x*_2_} is a geometrical co-ordinates and φ*_e_* (V) is an electric potential, while the external heat generation *Q_e_^ext^*(*x*_1_, *x*_2_) due to the electric heating for healthy tissue and tumor is defined as follows:

(2) $$Q_{e}^{ext}\left(x_{1},x_{2}\right)=\frac{\sigma_{e}}{2}{\left|E_{e}\left(x_{1},x_{2}\right)\right|}^{2}=\frac{\sigma_{e}}{2}{\left(\frac{\partial \varphi_{e}\left(x_{1},x_{2}\right)}{\partial x_{1}}+\frac{\partial \varphi_{e}\left(x_{1},x_{2}\right)}{\partial x_{2}}\right)}^{2},$$

where σ*_e_* (S/m) is an electrical conductivity.

The electric potential $\varphi_{e}\left(x_{1},x_{2}\right)$ inside the breast, cancer, and internal applicator is described by the system of Laplace equations [16,19,21,31]:

(3) $$\begin{array}{cc} x_{1},x_{2}\in \Omega_{e}: & \sigma_{e}\nabla^{2}\varphi_{e}\left(x_{1},x_{2}\right)=0 \end{array}.$$

For a heat transfer process in biological tissue, the Pennes model has been proposed e.g., [10,12,14,16,17,19,21,32,33,34,35]

(4) $$\begin{array}{c} x_{1},x_{2}\in \Omega_{e}: \end{array}\;\;\;\begin{array}{c} c_{e}\rho_{e}\frac{\partial T_{e}\left(x_{1},x_{2},t\right)}{\partial t}=\nabla \lambda_{e}\left(T\right)\nabla T_{e}\left(x_{1},x_{2},t\right)+k_{e}\left[T_{B}-T_{e}\left(x_{1},x_{2},t\right)\right]+Q_{met\;e}+Q_{e}^{ext} \end{array}\left(x_{1},x_{2}\right),$$

where *t* (s) denotes time, ρ*_e_* (kg/m^3^) is a density, *c_e_* (J/(kgK)) is a specific heat, λ*_e_* (W/(mK)) is a thermal conductivity, *T_e_* (K) is a temperature, *k_e_* = *G_Be_c_B_*ρ*_B_* (W/(m^3^K)) is a perfusion rate (*G_Be_* (1/s) is a blood perfusion coefficient, *c_B_* (J/(m^3^K)) is a specific heat of blood, ρ*_B_* (kg/m^3^) is a blood density), *T_B_* is a supplying arterial blood temperature (baseline physiological temperature), and *Q_met e_* (W/m^3^) is a metabolic heat source. The material properties used in calculations are collected in Table 1, based on [8]. At this point, it should be mentioned that the blood perfusion coefficient *G_B_* is a key parameter in the calculation of the bioheat transfer problem. The value of this parameter is different for different types of tissues (e.g., fat, skin, liver, heart, kidney, etc.) and increases by an order of magnitude in conditions different from the natural state (e.g., tumor).

The electrical and thermal conductivity are assumed as linear functions of temperature, approximated by Equations (5) and (6), respectively, for both healthy and tumor tissues [8,36]

(5) $$\sigma \left(T\right)=\sigma_{0}\left[1+0.02\left(T-T_{B}\right)\right]$$

(6) $$\lambda \left(T\right)=\lambda_{0}+0.0013\left(T-T_{B}\right)$$

where σ_0_ and λ_0_ are the baseline electrical and thermal conductivities, respectively, at baseline physiological temperature, *T_B_* = 37 °C (see: Table 1).

**Figure 3:**
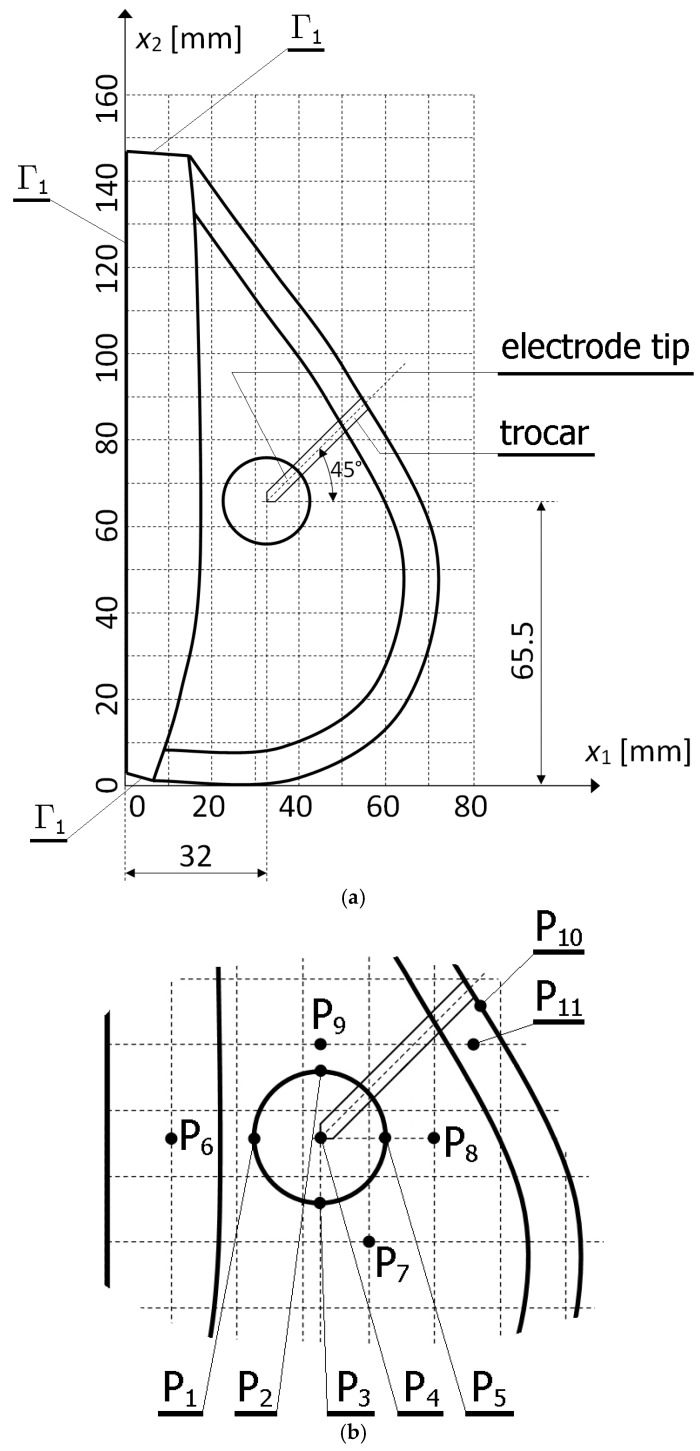
(**a**) Dimensions of the domain considered, position of tumor center and location of the internal applicator; (**b**) fragment of the analyzed geometry with the marked control points.

Differential equations which describe the electric (cf. Euqation (3)) and temperature (cf. Equation (4)) fields are supplemented by appropriate boundary conditions. Constant voltage boundary condition φ = *U* (*U* (V) is an electric potential of the electrode) is applied on the active part of the electrode, on the boundaries Γ_1_ (see: Figure 3) (simulating a ground pad) the zero volts (*U* = 0) is considered. On the remaining boundaries, an electric insulation is assumed. In the case of temperature field, on the boundaries Γ_1_, the body core temperature *T_b_* = 37 °C is assumed. The outer surface of breast is assumed to be subjected to natural convection

(7) $$-\lambda \frac{\partial T\left(x_{1},\;\;x_{2},\;\;t\right)}{\partial n}=\alpha_{eff}\left[T\left(x_{1},x_{2},\;\;t\right)-T_{amb}\right]$$

where α*_eff_* = 13.5 (W/(m^2^K)) represents the combined effective heat transfer coefficient due to convection, radiation, and evaporation [8,30,37,38], while the *T_amb_* = 25 °C is an ambient temperature. On the contact surfaces between breast sub-regions and tumor, the ideal electric and thermal contacts are assumed. Equation (4) is also supplemented by the initial conditions:

- the initial temperature of the electrode tine and trocar domains (indicated as Ω_5_ and Ω_6_) have been set to 25 °C, simulating the ambient room temperature condition
  (8) $$\begin{array}{cc} x_{1},x_{2}\in \Omega_{5}\cup \Omega_{6}: & {T\left(x_{1},x_{2},t\right)=T_{5-6}|}_{t=0} \end{array}=25\;{}^{\circ}C$$
- the initial temperature of the breast and tumor domains (indicated as Ω_1,_ Ω_2_, Ω_3_ and Ω_4_) have been considered to be uniform and the same as the body core temperature of the human body
  (9) $$\begin{array}{cc} x_{1},x_{2}\in \Omega_{1}\cup \Omega_{2}\cup \Omega_{3}\cup \Omega_{4}: & {T\left(x_{1},x_{2},t\right)=T_{1-4}|}_{t=0} \end{array}=37\;{}^{\circ}C$$

### 2.4. Arrhenius Scheme—A Model of Tissue Destruction

To estimate the degree of tissue destruction the Arrhenius integral, which describes the relationship between temperature and tissue damage, is used [39,40,41,42,43,44], where the relation between the tissue death with the temperature and the treatment time is given by

(10) $$Arr(x_{1},x_{2},\;\;t)=\int_{0}^{t^{f}}A\exp\left[-\frac{\Delta E}{RT\left(x_{1},x_{2},\;\;t\right)}\right]dt,$$

where *R* (J/(molK)) is a universal gas constant (*R* = 8.3143), Δ*E* (J/mol) is an activation energy, *A* (1/s) is a pre-exponential factor, *T*(*x*_1_*, x*_2_, *t*) denotes tissue temperature at the point {x_1_, x_2_}, and time *t ^f^*, while [0, *t^f^*] is a considered time interval. In the Arrhenius equation, the activation energy Δ*E* is defined as the minimum energy required to initiate the chemical reaction. The parameters *A* and Δ*E* are determined experimentally. For example, Table 2 summarizes the values of the above parameters for different types of tissues [45,46].

The main assumption of the Arrhenius scheme is that the thermal tissue damage is irreversible and total. It is assumed that irreversible tissue damage occurred, if

(11) $$Arr(x_{1},x_{2},t^{f})\geq 1$$

and the degree of tissue destruction as a result of thermal interaction corresponds to the share of damaged cells in the total volume of tissue *V_D_*, which is expressed by

(12) $$V_{D}(x_{1},x_{2},t)=\frac{\kappa_{0}-\kappa \left(t\right)}{\kappa_{0}}=1-\exp\left[-Arr\left(x_{1},x_{2},t\right)\right],$$

where κ_0_ is the initial concentration of healthy cells, whereas κ(*t*) the concentration of healthy cells after time *t* (*t*—thermal exposure time). Based on the knowledge of cell concentration, the relationship that defines the degree of tissue damage is written as follows:

(13) $$-Arr(x_{1},x_{2},t)=\ln\left(\frac{\kappa \left(t\right)}{\kappa_{0}}\right).$$

In computational practice. it is more convenient to use the integral relationship (10). According to the relationship (12), for the threshold value *Arr* = 1, 63% of the cells are permanently and irreversibly damaged (thermally damaged). It should be noted that, for the value *Arr* = 4.6, the degree of tissue damage is equal to 99%.

In addition, the value of perfusion coefficient *G_Be_* (cf. Equation (4)) depends on the value of the Arrhenius integral. The equation describing the relationship between tissue damage should reproduce the initial increase in perfusion caused by hyperemia (vasolidation), and then its fall due to collapse of vasculature (closing the vessels). Therefore, according to the literature [8,33,39], the perfusion coefficient can be taken as the following function:

(14) $$G_{Be}\left(Arr\right)=\{\begin{array}{ll} G_{Be}^{0}, & \mathrm{for}\;\;Arr=0\\ \left(1+25Arr-260Arr^{2}\right)G_{Be}^{0}, & \mathrm{for}\;\;0<Arr\leq 0.1\\ \left(1-Arr\right)G_{Be}^{0}, & \mathrm{for}\;\;0.1<Arr\leq 1\\ 0, & \mathrm{for}\;\;Arr>1 \end{array},$$

where *G_Be_*^0^ is the baseline blood perfusion coefficient (cf. Table 1).

## 3. Results

A heterogeneous (multi-layered) two-dimensional model of woman breast (see: Figure 2b) has been considered, in which a tumor of 2 cm diameter has been embedded. Dimensions of the domain considered, position of tumor center, and the position of internal applicator are presented in Figure 3a, while Figure 3b shows a fragment of the analyzed geometry with the marked control points. Table 3 contains the coordinates of the control points. The applicator consists of a stainless steel trocar and the nickel-titanium electrode tine. The applicator is located in the tumor area at an angle of 45° and its tip is in the center of the tumor. The electrical and thermophysical parameters of breast sub-regions, cancer, blood, electrode tip, and trocar are collected in Table 1, while the other parameters used for the calculations, discussed in the text, are collected in Table 4.

In order to solve the equations describing the potential of electric and temperature fields, the finite element method is applied using commercial software MSC MARC/MENTAT 2019 (64-bit). Bearing in mind a good representation of the geometry, and due to the expected large electrical and thermal gradients, the following mesh parameters were adopted: mesh density is equal to 17,704 quadrilateral, four-node elements. The computations have been performed using the personal workstation with a processor Intel^®^ Core^™^ i7 CPU 950 @3.07 GHz with 12 GB RAM.

**Figure 4:**
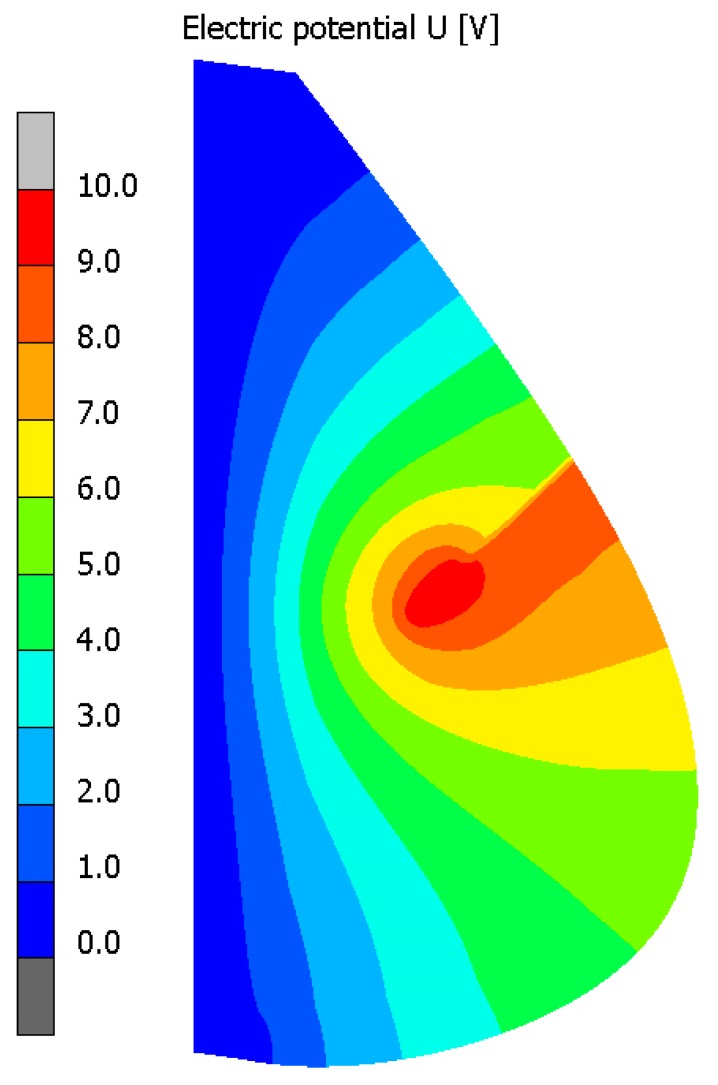
Electric potential distribution (*U* = 10V) (numerical calculations).

**Figure 5:**
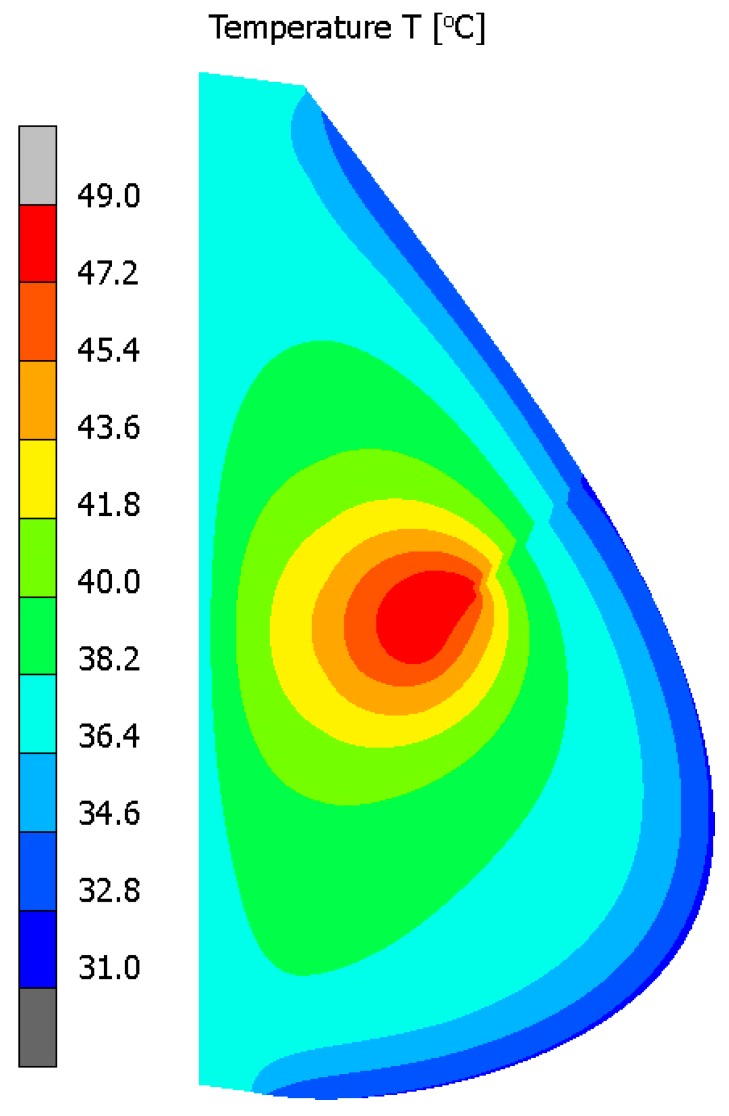
Temperature distribution after 10 min (*U* = 10 V) (numerical calculations).

**Figure 6:**
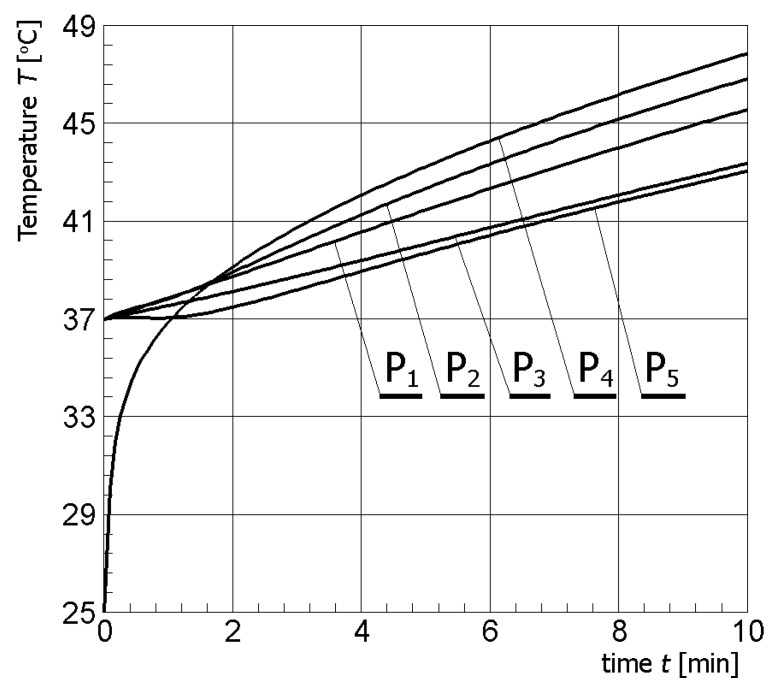
Temperature history at the control points in tumor (*U* = 10 V) (numerical calculations).

**Figure 7:**
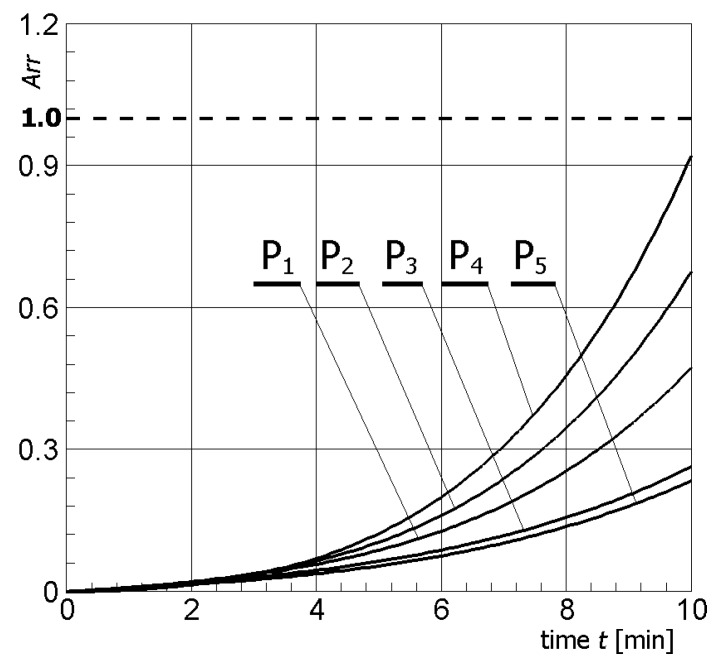
Arrhenius integral courses at the control points in tumor (*U* = 10 V).

**Figure 8:**
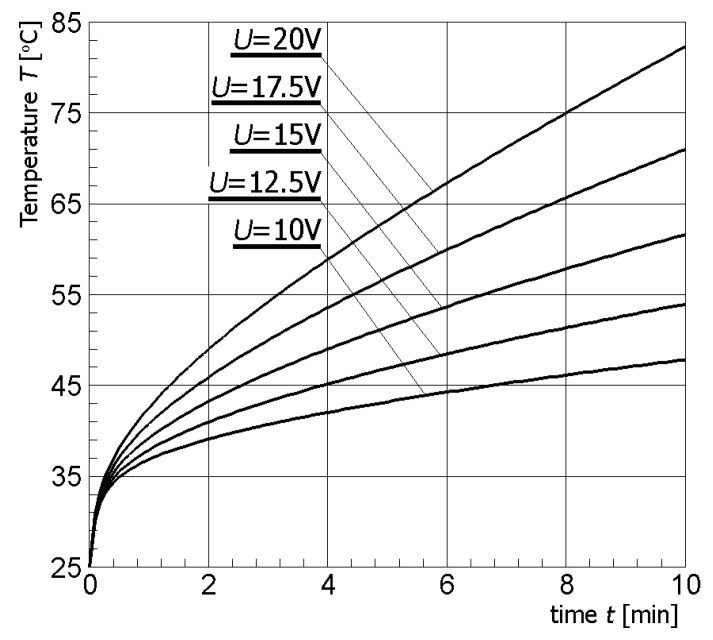
Temperature history at the control point P_4_ for different values of electric potential *U* (numerical calculations).

**Figure 9:**
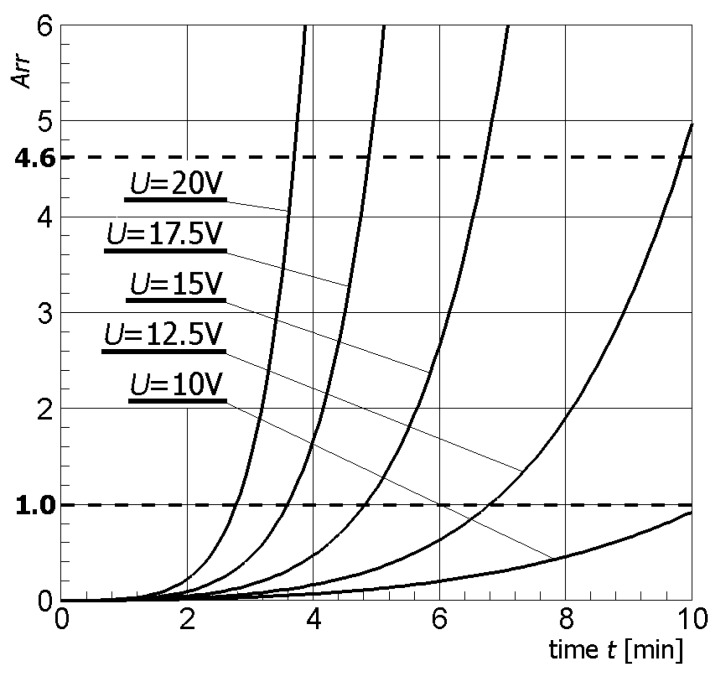
Arrhenius integral courses at the control point *P*_4_ for different values of electric potential *U*.

Figure 4 illustrates contour plots of electric potential for the exemplary voltage *U* = 10V, while Figure 5 shows the temperature distribution after 10 min during ablation heating. In Figure 6, the temperature history at the control points *P_i_* (*i* = 1, 2, …, 5) (cf. Figure 3b), obtained for the electric potential *U* = 10V and exposure time equal to 10 min, are shown, while, in Figure 7, the Arrhenius integral courses at the control points are presented. The contour maps presented in Figure 4 and Figure 5 and heating curves (see: Figure 6) were obtained on the basis of numerical calculations. In Figure 8, the temperature history for different values of electric potential *U* = {10, 12.5, 15, 17.5, 20}V at a control point *P*_1_ are presented, while, in Figure 9, the Arrhenius integral courses at a control point P_1_ are shown. The exposure time is also equal to 10 min. 

**Figure 10:**
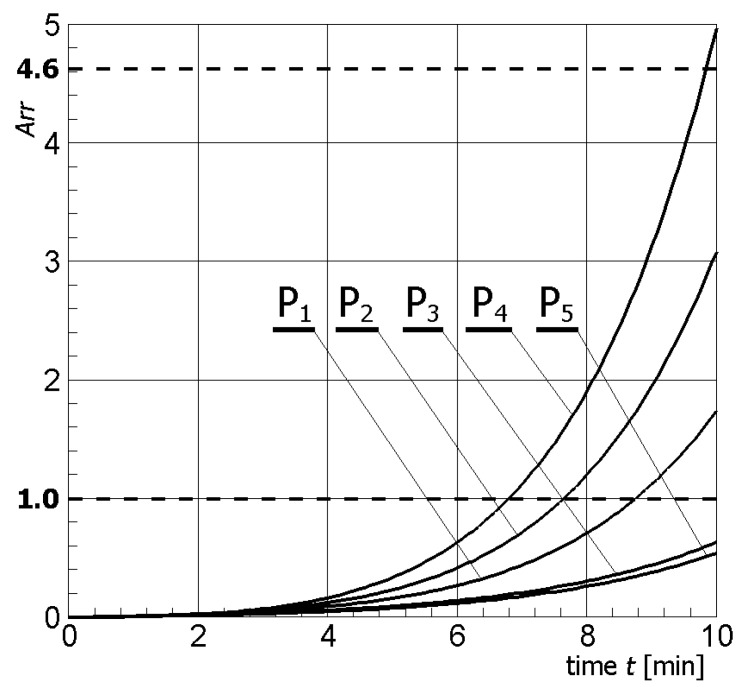
Arrhenius integral courses at the control points in tumors for *U* = 12.5 V.

As can be seen in Figure 9, the voltage adopted at 12.5 V causes the Arrhenius integral to assume a value exceeding 4.6 at control point P_4_ (probability of tissue destruction equal to 99%). Therefore, Figure 10 shows the value of the Arrhenius integral for all control points for an assumed voltage of 12.5 V.

**Figure 11:**
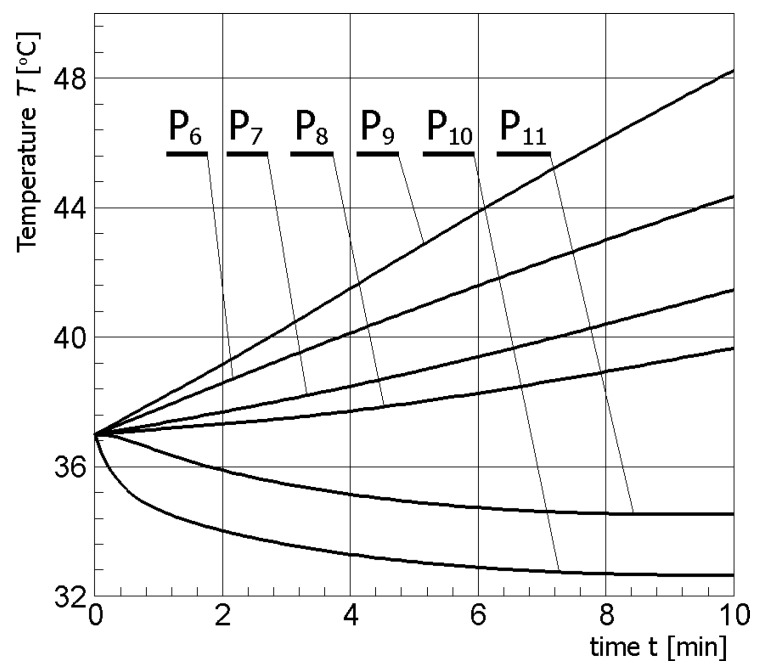
Temperature history at the control points (*U* = 12.5 V) (numerical calculations).

**Figure 12:**
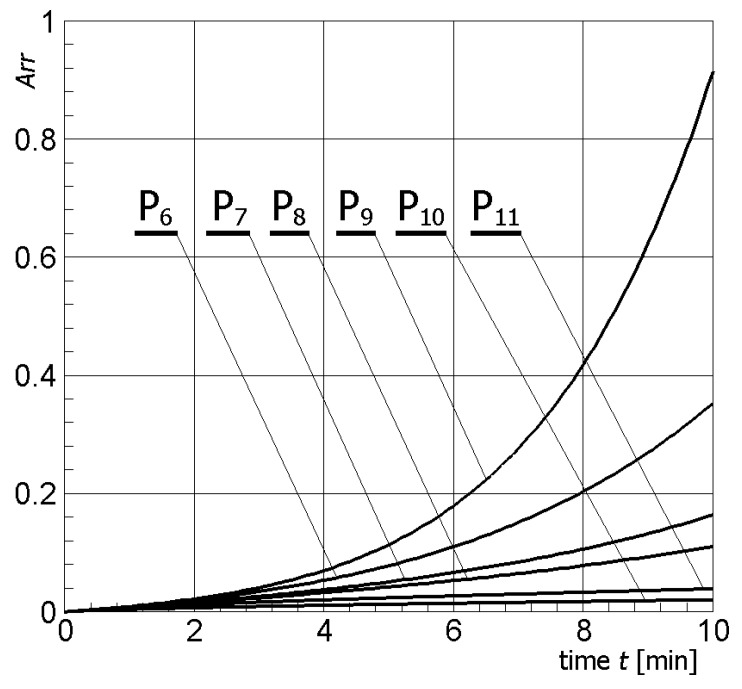
Arrhenius integral courses at the control points for *U* = 12.5 V.

In Figure 11, the temperature history at the control points *P_i_* (*i* = 6, 7, …, 11) (cf. Figure 3b), obtained for the electric potential *U* = 12.5 V and exposure time equal to 10 min, are shown, while, in Figure 12, the Arrhenius integral courses at these control points are presented.

Table 5 presents the value of the Arrhenius integral (cf. Figure 10), the probability of tissue destruction based on the formula (12), and temperature values for all control points after 10 min.

## 4. Discussion

Numerical modeling of the thermoablation process taking into account the influence of the electric field gives very good and promising results. Computer software using FEM allows simulation of the electro-thermal coupling process, and thus a simulation of breast tumor destruction using an internal applicator generating an electric field and causing the effect of thermal ablation. The mathematical model supplemented by the Arrhenius integral makes it possible to estimate the degree of destruction of cancerous tissue under the influence of high temperature.

As shown in the calculation results, the increase of electrical potential caused an increase of temperature within the analyzed area (cf. Figure 8). At the same time, an increase of temperature caused an increase of the value of the Arrhenius integral (cf. Figure 9).

In Figure 7, which shows the course of the Arrhenius integral for selected control points (see: Figure 3b), it can be observed that, only for point P_4_, the value of the *Arr* parameter approaches the value of the damage criterion (cf. Equation (11)). For the other control points, i.e., P_1_, P_2_, P_3_ and P_5_, the value resulting from Equation (10) is definitely less than 1.

The effect caused by the increase of the value of the electric potential on the active part of the electrode is shown in Figure 8 and Figure 9. The increase of electrical potential had a direct impact on the temperature rise and exposure time needed to obtain an Arrhenius integral value equal to 1.0 or 4.6. Analyzing Figure 9, it can be concluded that the optimal solution seems to be the choice of voltage equal to 12.5 V or 15 V, taking into account both the exposure time and the value of the Arrhenius integral. After about 10 min, tissue destruction determined by the location of the P_4_ control point can be seen with a 99% probability for *U* = 12.5 V, and after about 6.5 min for *U* = 15 V—(*Arr* > 4.6). If the potential value increased further, the Arrhenius integral increased very rapidly, which could also cause the destruction of healthy tissue surrounding the tumor.

Subsequent numerical simulations confirmed the above conclusions. Figure 10 shows the course of the Arrhenius integral at control points in the tumor for a voltage of 12.5 V. For points P_1_, P_2_, and, P_4_, the probability of destruction exceeds 80% (cf. Table 5), which corresponds to the temperature values obtained during the simulation. Of course, the exposure time to high temperature is also important.

The introduction of additional control points in healthy tissue, i.e., *P*_6_ (muscle), *P*_7_–*P*_9_ (gland), *P*_10_ (on the skin surface), and *P*_11_ (fat) allowed for numerical calculation of heating/cooling curves at these points (cf. Figure 11) and estimating the degree of tissue damage based on the Arrhenius integral (cf. Figure 12). Based on numerical simulations, it can be stated that the voltage selection on the active part of the electrode, equal to 12.5 V, was carried out correctly. The criterion of destruction (cf. Equation (11)) was not exceeded at any of the additional points analyzed. Only at point *P*_9_ located in the glandular tissue above the tumor can it be observed that the Arrhenius integral value is close to 1.0 (probability of destruction about 60%). However, this point is located about 5 mm from the tumor tissue, and it can be assumed that it is an acceptable region. 

The concentration of thermal energy in a specific area can be achieved by introducing paramagnetic nanoparticles into the tumor, e.g., [12,21,33], although such a procedure will cause an increase of temperature at the nanoparticle injection site but will not prevent possible damage to healthy tissue. An interesting solution seems to be the introduction into the tissue surrounding the tumor, nanoparticles with such thermophysical parameters that could constitute a kind of barrier against temperature increase, and thus protect healthy tissue from overheating. This issue will be analyzed in the future.

From the point of view of the possibility of supporting thermotherapeutic techniques, it is important to examine the impact of individual parameters occurring in the mathematical model on the degree of tissue destruction. Therefore, it is necessary to estimate, based on the sensitivity analysis methods, which parameter has the greatest effect on the computations’ results [47,48]. The results of calculations related to the sensitivity analysis were presented at the 4th Polish Congress of Mechanics and 23rd International Conference on Computer Methods in Mechanics [49].

**Figure 13:**
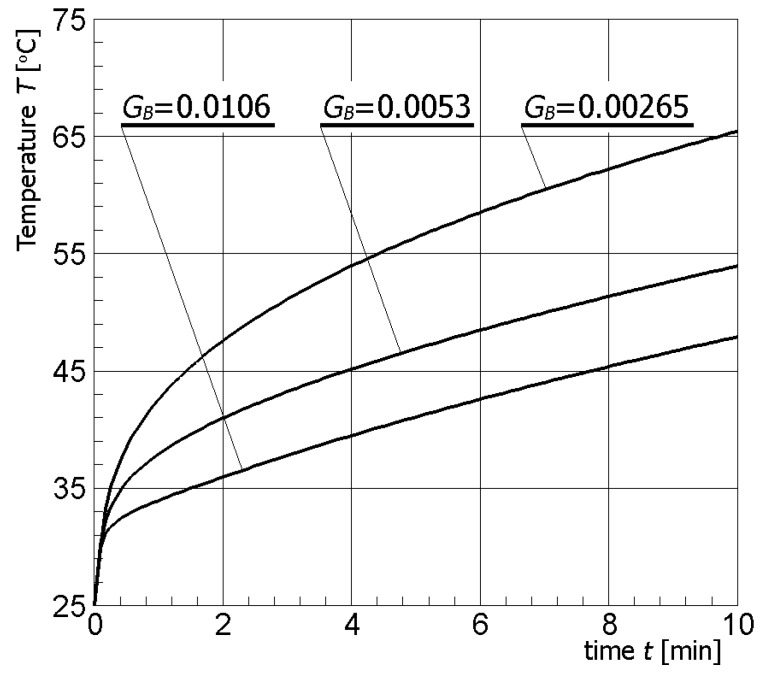
Temperature history at the control point *P*_4_ for different values of tumor perfusion coefficient.

**Figure 14:**
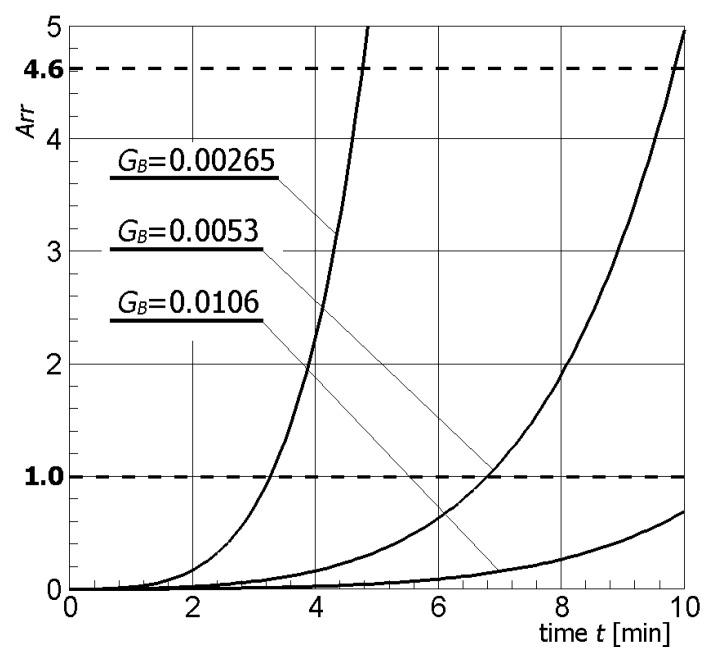
Arrhenius integral courses at the control point *P*_4_ for different values of tumor perfusion coefficient.

One of the important parameters of the mathematical model of bioheat transfer is undoubtedly the perfusion coefficient because the flowing blood acts as a heat sink. The level of tumor perfusion varies drastically depending on the type of tumor, and the value *G_B_* = 0.0053 1/s used for calculations (cf. Table 1) is only one of the possibilities [8,50]. Therefore, it is important to examine the impact of this parameter on the obtained results of calculations. For this purpose, different values of perfusion coefficient appropriate for normoperfusion (*G_B_* = 0.0053 1/s), hypoperfusion (*G_B_* = 0.00265 1/s), and hyperperfusion (*G_B_* = 0.0106 1/s) were adopted. The above values for hypo- and hyperperfusion were obtained by twice decreasing and increasing the reference value of the *G_B_* parameter, respectively. Figure 13 and Figure 14 present the temperature history and the course of the Arrhenius integral at point *P*_4_ (cf. Figure 3b), in which, due to its location, the changes are best observed. 

As could be expected, an increase of the perfusion coefficient caused a decrease of temperature during the numerical simulation of the thermoablation phenomenon (see Figure 13). The opposite situation occurred when the *G_B_* coefficient decreased and then the temperature increased. Changes in temperature distribution (cf. Figure 13) are also reflected in the course of the Arrhenius integral (cf. Figure 14). The above simulations indicate the importance of proper selection of parameters values occurring in the mathematical model.

## Figures and Tables

**Table 1:** Electrical and thermo-physical properties.

Tissue	Electrical Conductivity σ (S/m)	Specific Heat *c* (J/(kg·K))	Thermal Conductivity λ (W/(mK))	Density ρ (kg/m^3^)	Metabolic Heat Source *Q_met_* (W/m^3^)	Blood Perfusion *G_B_* (1/s)
Gland	0.563	2960	0.33	1041	700	0.0005
Fat	0.0254	2348	0.21	911	400	0.0002
Muscle	0.439	3421	0.49	1090	700	0.0008
Tumor	0.79	3770	0.48	1050	7792	0.0053
Blood Electrode Trocar	- 10^8^ 10^−^^5^	3617 840 1045	- 18 0.026	1050 6450 70	- - -	- - -

**Table 2:** The parameters *A* and Δ*E* for different tissue types.

Tissue Type	A (1/s)	Δ*E* (J/mol)
Breast	1.18 × 10^44^	3.02 × 10^5^
Liver	7.39 × 10^39^	2.58 × 10^5^
Skin	1.80 × 10^51^	3.27 × 10^5^
Tissue with the capillaries	1.98 × 10^106^	6.67 × 10^5^
Aorta	5.60 × 10^63^	4.30 × 10^5^

**Table 3:** Coordinates of control points.

Control Point	*x* (m)	*y* (m)
P_1_ (in tumor)	0.022	0.0655
P_2_ (in tumor)	0.032	0.0755
P_3_ (in tumor)	0.032	0.0555
P_4_ (in tumor)	0.032	0.0655
P_5_ (in tumor)	0.042	0.0655
P_6_ (in muscle)	0.01	0.0655
P_7_ (in gland)	0.04	0.05
P_8_ (in gland)	0.05	0.0655
P_9_ (in gland)	0.032	0.08
P_10_ (on the skin)	0.0575	0.086
P_11_ (in fat)	0.0565	0.08

**Table 4:** Values of parameters used during the numerical simulation.

Parameter	Value
Baseline physiological temperature *T_B_* Body core temperature *T_b_*	37 °C 37 °C
Initial temperature of the breast and tumor *T*_1–4_ Initial temperature of the electrode and trocar *T*_5–6_	37 °C 25 °C
Ambient temperature *T_amb_*	25 °C
Effective heat transfer coefficient α*_eff_*	13.5 W/(m^2^K)
Time step Δ*t* Length of the active part of the applicator Width of the applicator	1 s 10 mm 1.41 mm

**Table 5:** Arrhenius integral value and probability of tissue destruction.

Control Point	*Arr*	*T* °C	Probability of Destruction %
P_1_	1.7388	50.6	82.43
P_2_	3.0849	52.5	95.43
P_3_	0.6313	46.9	46.81
P_4_	4.9728	53.9	99.31
P_5_	0.5393	46.4	41.68
P_6_	0.3533	44.4	29.76
P_7_	0.1642	41.5	15.14
P_8_	0.1109	39.7	10.5
P_9_	0.9155	48.3	59.97
P_10_	0.021	32.7	2.08
P_11_	0.0394	34.6	3.86
